# Real-Time System for Daily Modal Split Estimation and OD Matrices Generation Using IoT Data: A Case Study of Tartu City

**DOI:** 10.3390/s22083030

**Published:** 2022-04-15

**Authors:** Kaveh Khoshkhah, Mozhgan Pourmoradnasseri, Amnir Hadachi, Helen Tera, Jakob Mass, Erald Keshi, Shan Wu

**Affiliations:** ITS Lab, Institute of Computer Science, University of Tartu, Narva mnt 18, 51009 Tartu, Estonia; mozhgan.pourmoradnasseri@ut.ee (M.P.); helen.tera@ut.ee (H.T.); jakob.mass@ut.ee (J.M.); erald.keshi@ut.ee (E.K.); shan.wu@ut.ee (S.W.)

**Keywords:** modal split analysis, OD matrix, real-time systems, internet of things (IoT), SUMO simulation, mobility modeling

## Abstract

In recent years, we have witnessed the emergence of the implementation and integration of significant working solutions in transportation, especially within the smart city concept. A lot of cities in Europe and around the world support this initiative of making their cities smarter for enhanced mobility and a sustainable environment. In this paper, we present a case study of Tartu city, where we developed and designed a daily real-time system for extracting and performing a modal split analysis. Our web-based platform relied on an optimization approach for calibrating our simulation in order to perform the analysis with the use of real data streams from IoT devices installed around the city. The results obtained from our system demonstrated acceptable performance versus the quality of the available data source. In addition, our platform provides downloadable OD matrices for each mode of mobility for the community.

## 1. Introduction

The rapid growth of urbanization has resulted in greater mobility demand from citizens daily. Consequently, understanding human mobility in urban areas has been of interest to the public and private sectors. Several models have been developed and employed in theory and practice to support decision makers and urban planners in transportation modeling, forecasting, and improving the quality of service. Traditional models for describing urban mobility primarily rely on travel diaries, surveys, and census to make a static prediction of the traffic demand. Due to the complex nature of travel demand at different times of the day or in additional days of the week, traditional approaches cannot fully capture different dimensions of urban dynamics. Moreover, as with other aspects of modern societies, urban mobility is subject to instantaneous change because of various reasons, including the increasing rate of car ownership, emerging business opportunities such as Mobility as a Service (MaaS), and pandemic outbreaks.

Although cities must maintain an up-to-date monitoring system for depicting the changes in the commuting patterns of citizens, the data are often challenging to collect, maintain, and update regularly for a real-time system. Existing solutions usually require a high amount of input data and computational power. One challenging task in this area is maximizing the functionality with available, limited resources and validation methods.

A wide range of frameworks has been developed and applied in the literature and practice for modeling urban mobility. Macro-simulations based on the four-step model [1] have been used for transportation modeling, such as PTV Visum in the case study conducted in Palembang, Indonesia [2]. Despite the solid theoretical foundation of traditional models, they are not as effective for real-time applications and under congestion conditions [3]. At the other end of the spectrum, activity-based micro-simulation [4,5,6] models have aimed to trace all the movements in the network on a second-by-second basis. As an example of an activity-based integrated system for travel forecasting and micro-simulation, we can name TRANSIMS (TRansportation ANalysis SIMulation System) [7,8], developed by the US department of transportation, or SIMBA MOBi [9], developed by the Swiss Federal Railways. Such models can capture highly precise details and multi-modal commutes; nonetheless, they are data-hungry and computationally demanding for real-time applications [10].

The availability of IoT data and the increasing capacity of computational power have enabled us to move from aggregated and static models to dynamic ones with more details. Nevertheless, no model can describe all aspects of the complex dynamics of cities, and there is always a trade-off between having more information and the availability of resources. Hence, depending on the applications, some simplifications in modeling are unavoidable.

In this work, we designed and implemented an integrated, data-driven, real-time system for modeling trip-based mobility in the city of Tartu, Estonia (a real-time system for daily mobility analysis and modal split estimation in Tartu city: https://its.cs.ut.ee/modsplit (accessed on 15 March 2022)). Using the stream of data from various IoT devices managed by the city government, our framework derives hourly Origin–Destination (OD) matrices for each mode of transportation, including vehicle, public transport, walking, and cycling trips, between every pair of city districts.

The presented case study is a proof of concept for the design of a daily real-time system using data streams from different sensors; it performs a late fusion of the estimated OD matrices in order to provide modal shares that reveal the mode of transport used by travelers. The lab validation showed reasonable robustness and good quality of results through simulation. In visualization formats and OD matrices, the generated outputs provide a unique overview of city-wide mobility patterns with many details, such as the dominant modes of transport in each city district throughout the day. The output of our work provides valuable insight to the city government for monitoring and planning. Moreover, the scientific community can benefit from the publicly available data in various studies related to mobility, pollution, and urban planning.

The rest of the paper is organized as fallows. After a brief overview of the related works, we describe our study area in Section 3 and the input data sources for our platform in Section 4. In Section 5, we provide a high-level vision of the architecture of our system pipeline. Then, in Section 6, Section 7, Section 8 and Section 9, we describe the details of different packages for each transportation mode, and in Section 10, we present an overview of our system’s web dashboard. In Section 11, we explain the results of the lab and field validations, and in Section 12, we conclude and discuss future directions.

## 2. Literature Review

The fundamental approach for retrieving the commuting patterns in urban areas is extracting the travel demand between pairs of Traffic Analysis Zones (TAZs) at different times, which is represented as the OD matrices. The traditional method used for estimating the OD demand in urban areas is the four-step model [11], which employs land use and demographic data for trip generation and trip distribution steps, and surveys for demand sampling [12]. Due to the high cost of surveys and with the availability of traffic count data on the selected links, other statistical methods that utilize the traffic counts alone or in combination with other data sources have been developed to derive time-dependent OD flow estimations [13].

Dynamic OD matrices are key inputs in advanced traffic management and traffic information systems. The availability of different data sources enables researchers to develop new techniques for extracting time-dependent OD matrices. The traffic data collected by the inductive loops or other point sensors can be complemented by less common data sources such as Bluetooth data [14,15], mobile network data [16,17], or GPS traces [18].

The estimation of OD matrices for motorized vehicles using traffic count data has been well-studied in the literature, from different perspectives such as entropy maximization [19], maximum likelihood [20], bi-level optimization [3,18,21], or machine learning techniques [22]. The main idea in these studies is the generation of a realistic traffic scenario that minimizes the distance from the point measurements [23].

While private vehicles are still the dominant mode of transportation in urban areas, many cities have started to rethink the concept of commuting by shifting the focus in urban planning and design towards more sustainable modes of transportation such as public transport, cycling, and walking [24,25]. This transition has resulted in an increasing need to understand mobility patterns with other modes of transportation. With the availability of various data sources such as ticket validation or bike-sharing data, and more importantly, massive data from IoT devices, it is more feasible to capture the urban dynamics with different modalities. Although it is crucial, understanding the mobility patterns of active modes of transport are more challenging for many reasons, which include topology and the connectivity of the network [26] as well as human factors [27].

Estimating the OD matrices of bicycle trips adapts available methods in the literature, such as the four-step model [28] or the optimization approach [29] for calibration. However, due to the geographical characteristics of the region of study and based on the availability of input data, tailor-made solutions are required.

Extracting a dynamic, city-scale OD matrix for walking trips remains among the most challenging tasks in mobility modeling. Some studies characterize pedestrian dynamics based on statistical physics models [30] such as hydrodynamic, gas-kinetic, or social force models. However, these methods are not efficient in large-scale and complex environments. A few studies have approached the problem by combining the activity-based user equilibrium assignment model and bi-level optimization [31] for extracting the static OD matrix of pedestrians. With the increase in computational capacity in recent years, agent-based models for simulating pedestrian movements have been more popular [32,33]. However, the high demand for data and power makes micro-simulation models inefficient for city-scale studies.

Available large-scale case studies are conducted based on historical data, primarily for short time frames and predominantly for vehicles; for example, the estimation of the OD matrix of passenger cars during morning peak hours in Medellin, Colombia [19], or the 24 h semi-dynamic traffic assignment in the Chukyo metropolitan area, Japan [34] , or the day-long OD demand estimations in the Rabat region in Morocco [35].

While improving transportation and mobility plays a central role in smart, sustainable cities [36], when it comes to extracting urban mobility patterns for different modes of transport and in real-time, we witness a considerable gap in imperial studies. Besides methodological and computational shortcomings, maintaining a reliable input data stream is the main bottleneck in designing real-time, integrated transportation modeling systems. Thanks to our collaboration with the city of Tartu, which provided us with the required data, in this work, we try to close this gap by expanding the state-of-the-art algorithms and methods for delivering a dynamic model of urban mobility in the city. The result of our work captures the hourly OD flow of each mode of transportation and can be a valuable input for urban planners, policymakers, and researchers for further studies in the area of activity-based and agent-based transport modeling [37].

## 3. Study Area

**Figure 1:**
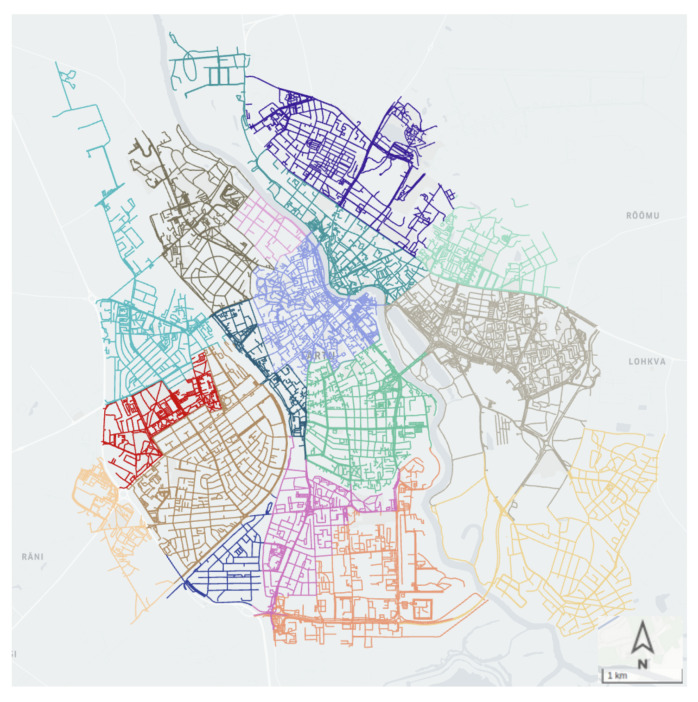
Road network of the city of Tartu. Each color represents an administrative district.

We implemented our methodology in Tartu, the second-largest city in Estonia. The city’s approximate land area is 39 km${}^{2}$, and its population is 91,000. The city is officially divided into 17 administrative districts, ranging from 0.5 km${}^{2}$ to 5.5 km${}^{2}$, with an average of 2.23 km${}^{2}$. We considered each district as a spatial unit in our study. Figure 1 shows the districts of the city of Tartu in different colors.

Several IoT sensors are installed and managed by the city of Tartu throughout the city for collecting various traffic information. Our methodology diligently relies on the non-aggregated extracted counts, which are explained in the following section.

## 4. Data Source

The data sources in our system are various, as demonstrated in Table 1. However, we can categorize them into two major types: dynamic data and static data. Dynamic sources represent stream data fetched on demand from the sensors each time the pipeline runs. Static data refers to geo-data used for building OD matrices and is expected to change infrequently, such as the geo-information about Tartu district borders.

### 4.1. Dynamic Source

In our case study, the IoT devices are handled by the Cumulocity Internet of things platform instance [38], and our dynamic sources unveil their data to the system via HTTPS RESTful API. Moreover, we used three different types of sensor counts (ECO, Thinnect, AVC) and one kind of coordinate information (Bus GTFS). The available sensors are designed for a specific type of counting, and they are as follows:

1. ECO sensors counting pedestrians and bicycles;
2. Thinnect sensors counting vehicles and light traffic (pedestrians and bicycles, undistinguished);
3. AVC sensors counting vehicles.

Furthermore, most of the sensors can consider in their counting the direction of the movement of the vehicles or humans (they count events for a particular direction of motion).

In addition, we have access to further information such as anonymous data of bus ticket validations thanks to the Ridango API (Ridango is a company providing transport solutions: www.ridango.com (accessed on 10 September 2021)). The validation events include information about the bus stop, the bus line, and the time of the event. The up-to-date bus stop information is obtained from Peatus (Peatus is a journey planner by the public transportation in Estonia: www.peatus.ee (accessed on 12 September 2021)). Additionally, data about individual bike trips with the city bike-sharing system are collected using the Bewegen API (Bewegen is a company providing and handling bike-sharing systems: www.bewegen.com (accessed on 16 September 2021)). This information also includes the time and bike station where the journey started and where it ended.

### 4.2. Static Source

Two sets of static data are used in our system design:

1. A file depicting the geographic shapes/borders of the districts used to identify which trip originated/ended in which district; the file also contains an assigned identifier code for each district (Figure 1).
2. A mapping of City Bike stations to the districts.

## 5. System Architecture

**Figure 2:**
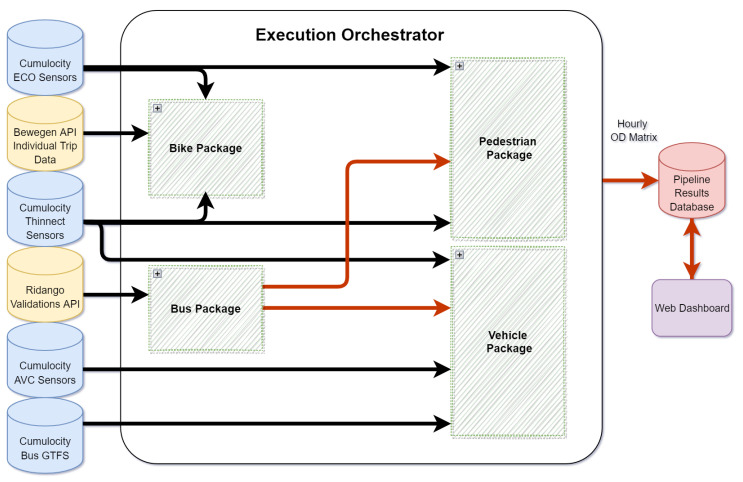
The proposed system architecture.

We designed our platform following the architecture demonstrated in Figure 2. The software solution proposed contains two main blocks: the data analysis pipeline and web dashboard. The data analysis pipeline extracts fine-grained data (usually single-trip or single sensor-level granularity) from several modality-specific data sources (e.g., the bus ticket validation info system).

The raw data are analyzed to produce hourly and district-level trip estimates for the entire city in the form of an OD matrix for each of the four transport modes and for a one-hour time frame.

The web dashboard provides a graphical user interface of the results in a web application with interactive visualization and browsing. Additionally, the dashboard offers further aggregation functionalities such as presenting results from a 24 h period based on the hourly pipeline outputs. The two blocks are linked by persistent storage to an SQL database for post evaluation and troubleshooting. The pipeline is scheduled to execute automatically once every 24 h to analyze the previous day’s information and store the results to make them available to the web dashboard. The system’s pipeline involves four packages for each transport modality and an execution orchestrator. The orchestrator ensures the execution of individual packages in a specified order and manages the re-execution of packages that produce failures.

Every modality-specific package follows some basic designed steps, and they are as follows:

1. Fetching all necessary input data for their execution (via HTTPS REST API);
2. Performing the aggregation and analysis as well as storage.

If one package’s input includes the results of another package (e.g., as in the case of a vehicle package relying on a bus package’s output), the package assumes the execution orchestrator to have already finished the execution of the necessary packages.

In the following sections, we describe the algorithms and details of each package.

## 6. Bicycle Package

**Figure 3:**
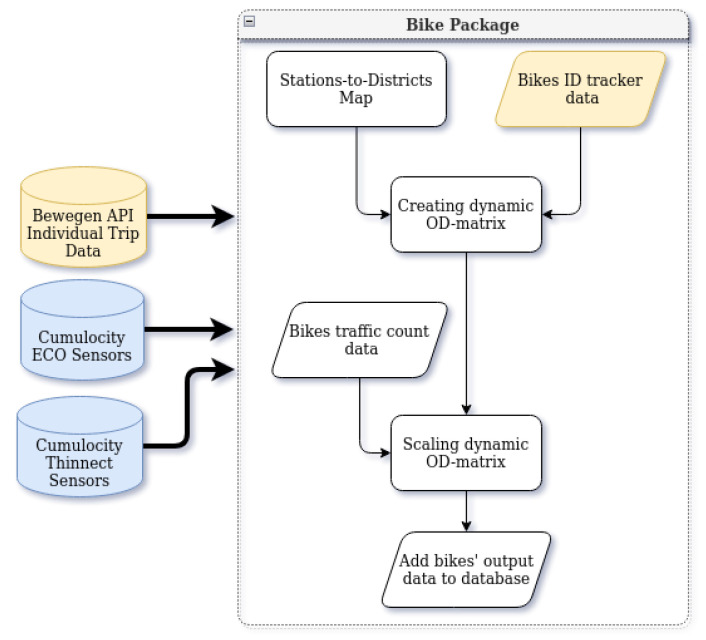
Bicycle package architecture.

This package aims to estimate the hourly number of bike trips between each pair of districts. The architecture of the estimation method is presented in Figure 3.

**Figure 4:**
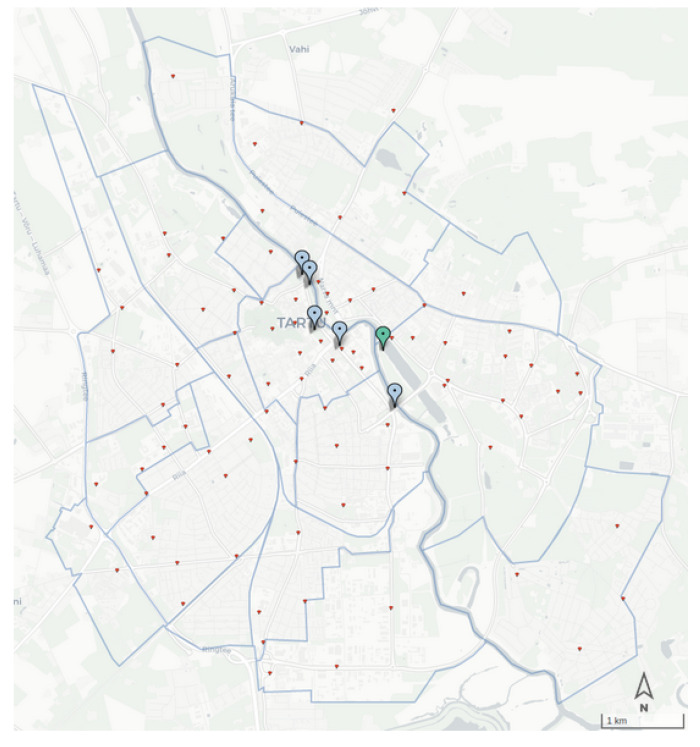
Data collection points for bike package: Thinnect sensors (blue), ECO sensor (green), and location of Tartu Smart Bike stations (red).

Besides the availability of sensor data in the Cumulocity dataset, the estimation takes into account the data of bike trips on Tartu’s bike-sharing system. *Tartu Smart Bike system* (https://ratas.tartu.ee (accessed on 12 September 2021)) is a popular self-service bike-sharing system that comprises 750 bikes and more than 80 stations in the city of Tartu. The station map of Tartu Smart Bike is presented in Figure 4.

We assume that the mobility pattern of the personal bikes follows a pattern similar to that of bike-sharing trips. Therefore, using sensor data, we calculate a ratio for scaling up the number of bike-sharing trips to the actual number of bike trips (including the personal bikes).

Thus, the first step is pulling the relevant data from the Bewegen API. The Bewegen API gives us the origin and destination stations for each bike trip with the exact time for the detachment and attachment of bikes at the stations.

The approach adopted to calculate the scaling ratio for our case study relies on the city’s topology. The city of Tartu is divided into two parts by the Emajõgi river. Seven bridges connect the two sides of the river. Among them, two bridges are only dedicated to crossing pedestrians and cyclists. Except for the Ihaste bridge that is located on the edge of the city, all bridges are covered by Thinnect or ECO sensors (Figure 4).

There are five Thinnect sensors, which count all the light road users (bikes and pedestrians combined), and one ECO sensor located at Turu bridge, which measures the cyclists and pedestrians separately. The ratio between cyclists and pedestrians crossing Turu bridge is used to determine the number of cyclists from among the light road users in the Thinnect sensor readings on the other bridge.

Next, following simple logic, the bike-sharing trips that cross any of the bridges are counted; if the origin and destination stations are on different sides of the river, the bike must pass one of the bridges and be included in the sensors’ counts. By using the total number of cyclists counted on all the bridges and the number of Tartu Smart Bike trips crossing any of the bridges, we are able to calculate the final share of city bike users out of the total bike users.

As the last step, the city bike data are parsed and scaled. Since the final solution is district-based, the station-level city bike data must be mapped to the district-level data. Once the mapping process is completed, the scaling factor is applied, and the data is saved as an OD matrix of trips between districts for every hour.

## 7. Bus Package

**Figure 5:**
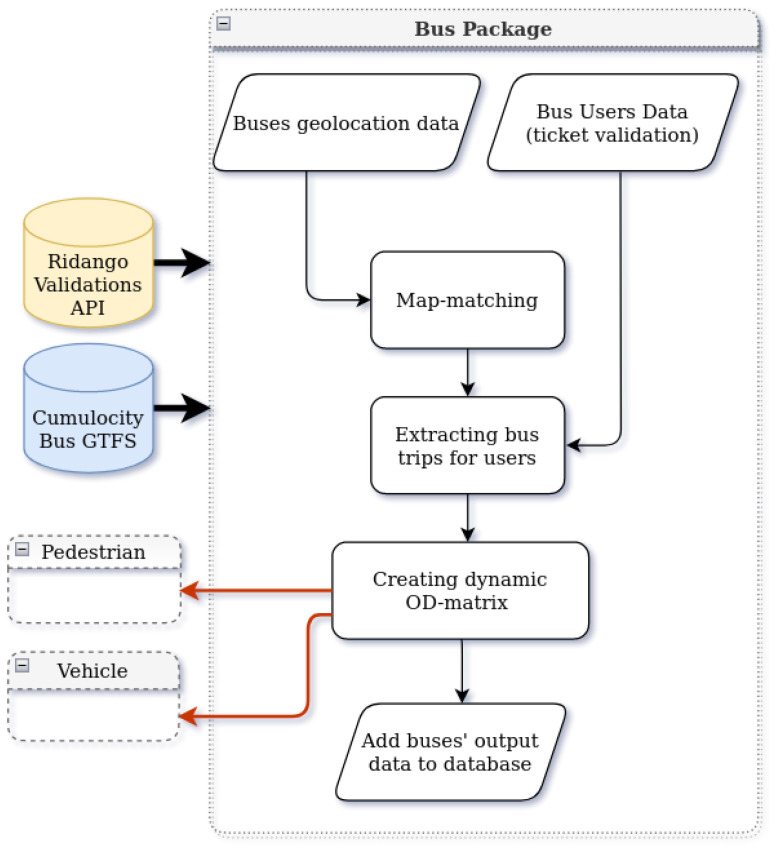
Bus package architecture.

Public transport in the city of Tartu includes seventeen inner-city bus lines. Since 2015, the ticketing system has switched to contactless chip cards or stickers that must be swiped at onboard validators upon entering a bus. The objective of the bus package is to estimate the district-level OD matrix of the bus passengers based on bus routes and validations tickets, assuming that all the passengers validate their tickets. The ticket validation process automatically registers the bus stop, representing each passenger’s location of origin. While there is no information about the locations where a passenger exits the bus, we estimate the trip destination for each passenger. The high-level architecture of the bus package is presented in Figure 5.

The approach used relies on three main steps. The first step is checking and processing the two sources of data. The GPS traces streamed by the bus are filtered and map-matched to remove incoherence or geo-location errors. The Ridango API provides daily information about city buses—their routes, trips, and passenger validations on each bus. Additionally, information about stops is extracted daily from Peatus.

In the second step, the destination of each bus passenger is estimated. We assume the probability that a station chosen to be a destination in the morning peak would be close to the probability that the same station chosen to be a origin station in the afternoon peak. In other words, people usually have regular round trips starting from the place of residence [39].

Given the origin station of a passenger, all the possible destinations on the same route are listed, excluding the stations where the bus does not stop. Then, we choose a destination from the list of potential destinations according to a probability distribution proportional to the opposite peak interval on the same day.

Finally, after having the bus passenger’s start and end stations, the stations are mapped to the city districts, and the hourly district-level OD matrices are formed. These matrices present the mobility flow of bus users in the city and will be used later as initialization input for pedestrian and vehicle packages.

## 8. Vehicles Package

**Figure 6:**
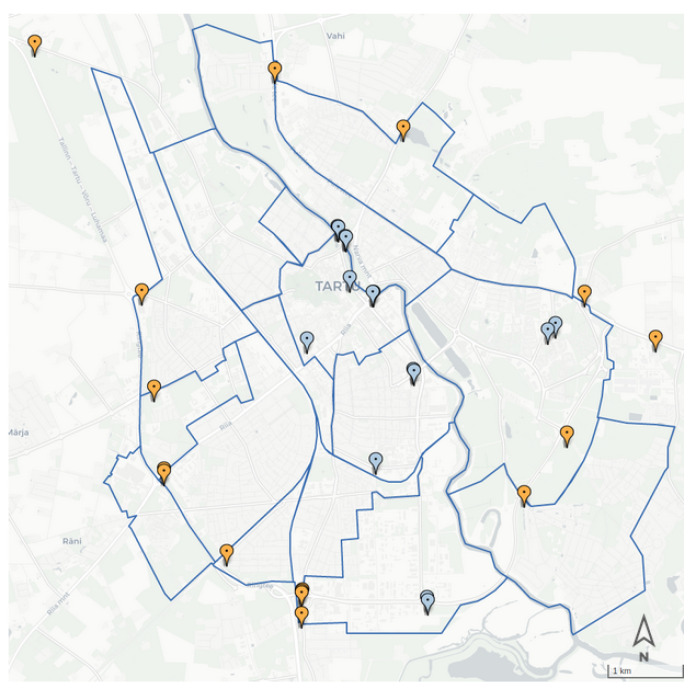
Locations of AVC Vehicle counters (orange) and Thinnect counters (blue).

Most cities employ traffic counters to understand the traffic volume in different city regions. Nonetheless, the counters do not cover all the city streets, and the sensors’ counts can be considered as a sample of the traffic dynamics. Figure 6 shows the location of the sensors in the city of Tartu.

In order to estimate the vehicle trips, we adopt off-the-shelf techniques in the literature for simulating urban trips with the use of a traffic simulator. In this work, Simulation of Urban Mobility (SUMO) is used [40]. SUMO (https://www.eclipse.org/sumo/ (accessed on 24 June 2021)) is an open-source, microscopic, and continuous traffic simulation utilized to overcome the gap between the sensors’ traffic counts on selected links and actual traffic flow in the network. Thanks to the calibration model, the system is capable of using the real counts to adjust the simulated traffic flow and represent it as close to reality as possible.

The vehicle package aims to make a daily estimation of vehicle trips within the city by performing a calibration step using data from IoT devices (Figure 6). The AVC sensors in a strategic location on the border of the city allow us to have a big picture of the vehicles entering and leaving the city of Tartu. In addition, Thinnect sensors provide us with a glimpse into the mobility pattern of inner-city areas.

**Figure 7:**
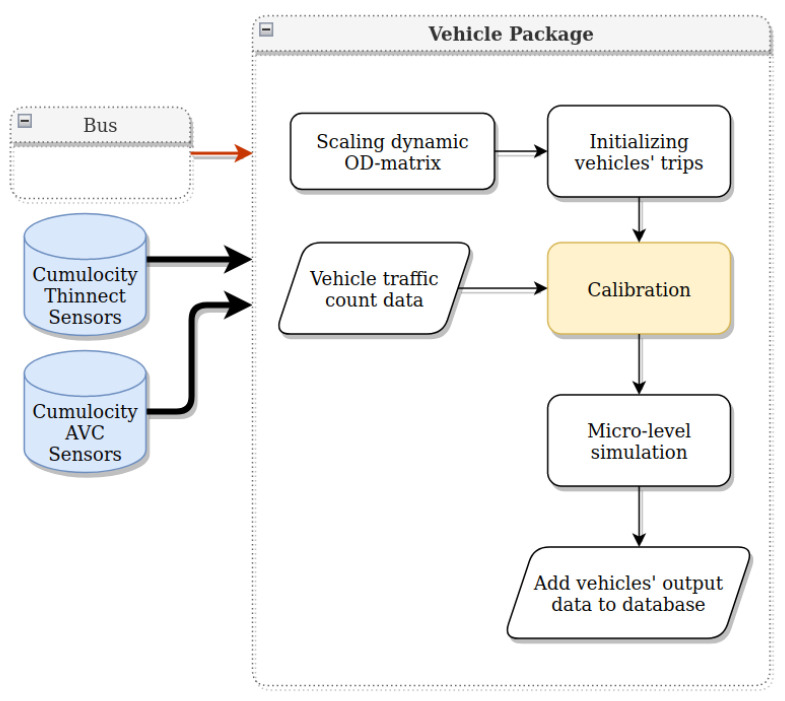
Vehicle package architecture.

The architecture of the vehicle package is presented in Figure 7.

In the first step, a map of the regions of interest is extracted from Open Street Map (OSM) (https://www.openstreetmap.org (accessed on 14 February 2022)) and converted for the SUMO network. For simulation initialization in SUMO, a set of trips acquired for 24 h is required. In approximating the initial OD matrix, we take into account two sets of inputs. First, the OD matrix of bus trips (bus package output) reflects a mobility pattern inside the city. Next, to cover the trips going into and out of the city, the data from AVC sensors and district populations are used. Lastly, sensor data are used to calibrate the number of trips. For calibration and traffic assignment, the Calibration of Dynamic Traffic Simulations (Cadyts) tool (https://sumo.dlr.de/docs/Contributed/Cadyts.html (accessed on 20 July 2021)) is used [41].

## 9. Pedestrian Package

Estimating foot traffic is the most challenging task in understanding urban dynamics. The common approach in the literature for estimating the share of pedestrians is through the use of survey data in the whole estimation [42] or as the base of estimation [43]. However, the surveys usually cover a small fraction of the population and are repeated with low frequency. In the absence of survey data for Tartu city, we developed a novel data-driven approach for estimating the hourly district-level OD matrix of foot trips in the city, using sparse pedestrian counters and assessing the topology of the walking network [44].

**Figure 8:**
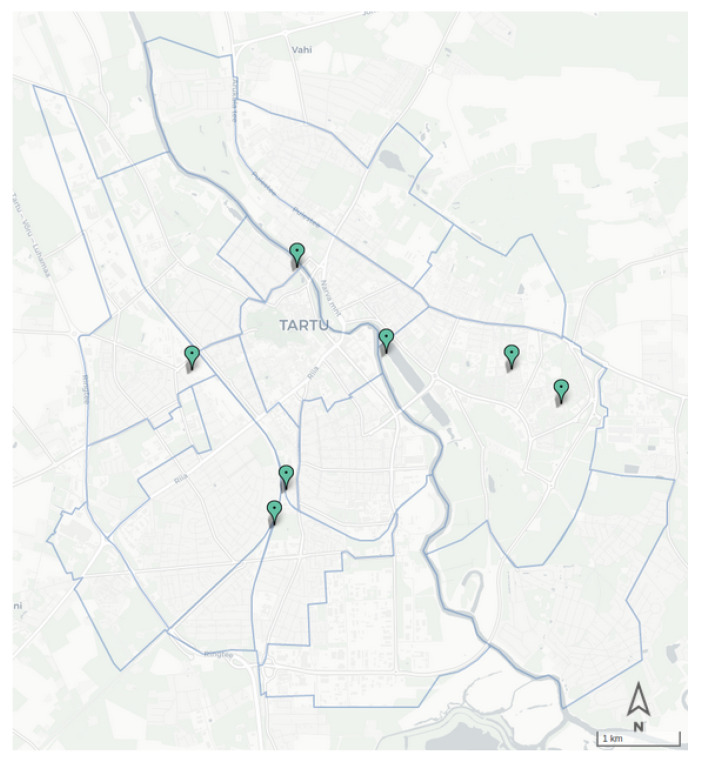
Locations of ECO sensors for pedestrian counts.

**Figure 9:**
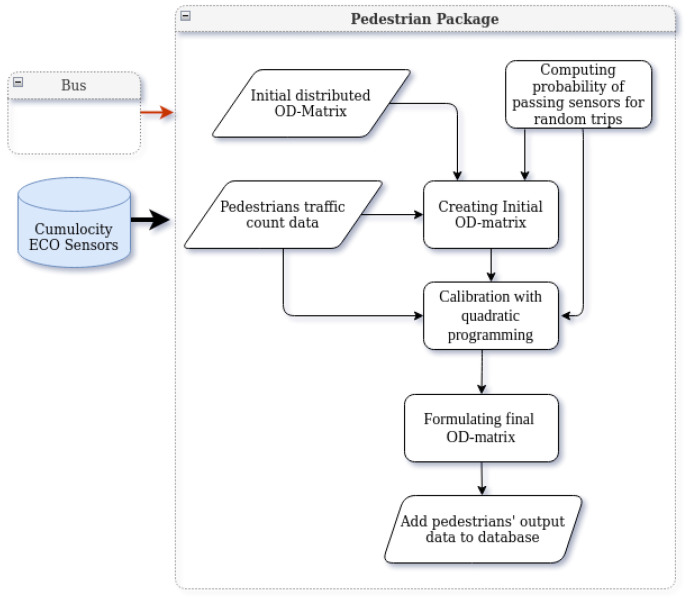
Pedestrian package architecture.

Figure 8 presents the location of ECO sensors utilized in the pedestrian package; the architecture of the package is presented in Figure 9. The solution relies on two sets of inputs; one is the foot traffic count from the Cumulocity database, and the other one is an initial OD matrix of the pedestrian trip distribution. The initial distribution is a matrix $D=(\delta_{ij})$, with the trip probability distribution between spatial units of the area of study $\sum_{ij}\delta_{ij}=1$. This matrix can be obtained from a variety of external information sources such as travel surveys or population data.

We assume that the pedestrian mobility pattern is similar to the bus passengers’ mobility to some extent. Therefore, for the initial OD matrix distribution D, we follow the probability distribution according to the bus OD matrix (the output of the bus package). Based on the distribution D, a set of random trips is generated. Moreover, to make our simulation as close to reality as possible, we removed very long journeys from the sets of trips. As a result, all trip distances are less than 3000 m. This gives us an initial OD matrix $\bar{X}$ based on D and the sensor data.

Our method for pedestrian flow estimation is as follows. For every pair of districts *i* and *j*, we computed the probability that a trip from *i* to *j* passes by the sensor *k*. Denote this probability with $a_{ij}^{\left(k\right)}$. This probability is computed based on the generation and simulation of many random trips between different city districts and by observing the share of the trips that pass by the sensor locations. This observation is highly affected by the topology of the network and its connectivity.

The objective is to estimate the number of trips between each pair of districts, such that the trips that pass through the sensors’ locations match the actual sensor reading. In other words, for every sensor *k*:

(1) $$\sum_{ij}a_{ij}^{\left(k\right)}x_{ij}=y^{\left(k\right)}$$

where $y^{\left(k\right)}$ is the number of pedestrians detected by sensor *k*, and $x_{ij}$ is the number of pedestrian trips between districts *i* and *j*.

At the same time, we aim to find a realistic OD matrix $X=(x_{ij})$, which means that it should not deviate very much from the initial OD matrix $\bar{X}$. To ensure this, we are interested in minimizing $∥X-\bar{X}{∥}_{2}$. Hence, the following quadratic programming problem is obtained:

(2) $$\begin{array}{ccc} \min & ∥X-\bar{X}{∥}_{2} & \\ \mathrm{subject}\;\mathrm{to} & \sum_{ij}a_{ij}^{\left(k\right)}x_{ij}=y^{\left(k\right)} & \\ \; & k=1,\cdots ,\mathrm{number}\;\mathrm{of}\;\mathrm{sensors},\\ \; & i,j=1,\cdots ,\mathrm{number}\;\mathrm{of}\;\mathrm{districts}. \end{array}$$

In order to solving the minimization problem, the optimization package QP Solvers for Python (https://github.com/stephane-caron/qpsolvers (accessed on 9 October 2021)) is used.

## 10. Web Dashboard

To allow for the exploration and visualization of the output of the data analysis pipeline, we developed a web-based dashboard application (https://its.cs.ut.ee/modsplit/ (accessed on 1 April 2022)). The NodeJS-based web application interfaces with the same database as that of the main pipeline. Users can browse results from different dates, see how a given date’s results compare to some historical averages, and export (download) the OD matrix data.

The web dashboard features three different perspectives: City-level summary, District Analysis, and Data Export. For each of these features, the user can select a single date, and the data for the selected date are presented according to the operational perspective.

### 10.1. City-Level Summary

**Figure 10:**
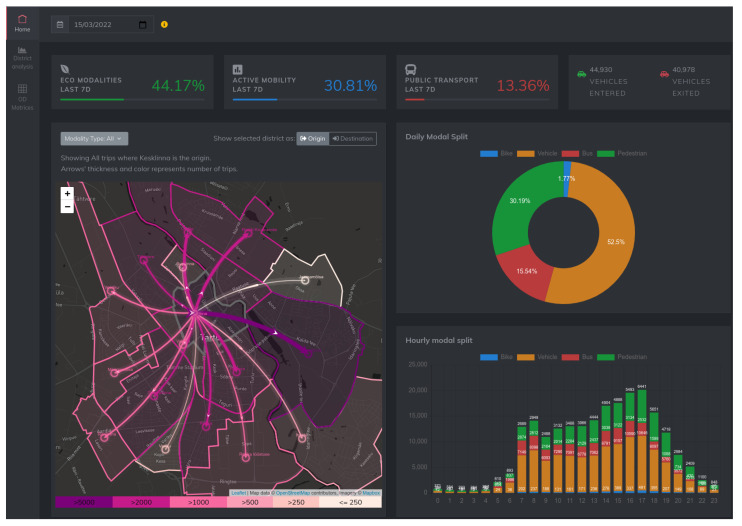
City-level summary perspective of the web dashboard.

This perspective acts as the *main* view of the dashboard, aimed at giving an overview of the situation at the city level. A screenshot of this perspective can be seen in Figure 10 The main features of this perspective are as follows:

1. **Map-based visualization** shows the number of commuters traveling to other districts, with a heat map-like color visualization over a geographical map for a selected district, with the possibility to select the modality type.
2. **Pie and bar charts** show a city-level modal split and a city-level hourly modal split, respectively, for the selected day.
3. **Aggregate values** show the average modal shares of public transport, active mobility, and eco-modality over the last seven days. These indicators help to monitor the mobility trends in the city.
4. **Number of vehicles that entered/exited** the city is measured based on the readings of AVC sensors, which cover all major roads crossing the border of the city, during the whole day.

### 10.2. District Analysis View

This perspective focuses on a single district to understand the popular origins/destinations in relation to the selected district and the overall modal share in the district. It includes the following features:

1. **Map-based visualization** is a map view that can be used to change the active district. For the selected district, the map shows the number of trips to other districts with a heat map-like colour visualization.
2. **Hourly bar chart** shows the hourly modal split for the selected district.
3. **District-specific OD matrix** presents the numerical values for each mode of transport for the selected district.

### 10.3. Data Export

This perspective shows five different tables displaying daily district-level OD matrices for each mode of transport and the aggregated OD matrix. Users can download each of the matrices as a CSV file. In addition, the back-end of the web application allows querying for district-level data of hourly OD matrices, with corresponding JSON-formatted responses.

## 11. Evaluation

The evaluation process adopted in our research work is divided into a lab experiment and a field test. In the lab evaluation, we generate random synthetic trips in the network and produce synthetic sensor data. Then, the synthetic data generated from our virtual sensors are used as input for our system. Finally, we compare the number of synthetic trips with each transport mode and with the output of each package.

To assess the functionality of the system in a real environment, it is essential to conduct large-scale testing, ideally by collecting reference data about the traffic modality in the whole city, which requires an enormous amount of resources. With the available resources, we planned a small field test in one district of Tartu—Supilinn—on 10 December 2021, from 08:00 to 10:00. Supilinn is the smallest district in the city, with controllable entrances/exits; nevertheless, it is the second most densely populated district in Tartu, with a population density of 4427/km${}^{2}$. We only compared the modal split computed from the manual counts with the modal split estimated by our system in the same time frame for the field evaluation.

The evaluation and analysis will exclude the bus package since the data are collected from the IoT devices, which reflect the trips accurately with the equivalent ticket validation. Hence, the results will be analyzed through the bicycle, vehicle, and pedestrian packages.

### 11.1. Bicycle Package

The Tartu Smart Bike data are the basis of the package calculations and are always assumed to be reliable. Therefore, the part of the bike package that needs validating is the calculation of the scaling ratio between the trips of shared city bikes and those of all bikes.

To run the bike package on the synthetic data, three relevant data sets are required:

a. City bike trip data, containing the station of origin and the destination;
b. Turu bridge ECO sensor data, containing separate counts of pedestrians and cyclists;
c. Thinnect sensor data on other bridges, containing combined counts of pedestrians and cyclists.

To validate the scaling ratio in the bike package, the following steps are performed.

1. A total of 2145 pedestrian trips, 1680 city bike trips, and 1391 normal bike trips are generated and simulated using the SUMO simulator;
2. The synthetic sensor readings for the virtual bridge sensors are extracted from the simulator;
3. The data are fed into the bike package;
4. The package computes the scaling ratio between the city bike trips and all bike trips.

Knowing the actual counts of personal and city bikes in the simulation, the result from the scaling solution could be compared to the existing bicycle count from the simulation.

The package computes with the scaling number of 1.922 (total bikes/city bikes) on the synthetic data. Given the number of city bike trips, the total estimated number of bike trips in the package can be calculated as follows:

(3) 1680×1.922=3229.

In comparison to the total number of bicycle trips from the generated data (1680+1391=3071), the relative error margin of the solution is calculated as follows:

(4) $$E=\frac{\mathrm{absolute}\;\mathrm{error}}{\mathrm{real}\;\mathrm{value}}=\frac{\left|3229-3071\right|}{3071}=5.16\%$$

### 11.2. Vehicle Package

The vehicle package consists of two main parts: the preparation of the initial OD matrix and the calibration method. The calibration method implemented in our work is performed by utilizing the Cadyts library, and we refer to [41,45] for the validation.

To understand the sensitivity of the vehicle package to the initial OD matrix, we run a test in the lab environment for a set of random synthetic trips. The package is fed, with the artificial sensor data of vehicle trips as an input; then, the output is compared with the synthetic trips. The lab validation for the vehicle package consists of the following steps:

1. The schedule of buses is given to the simulator;
2. A set of random vehicle trips are generated. In this step, a trip is a pair of origin and destination;
3. A set of synthetic multi-modal trips (bus and walk) is generated;
4. Trips are simulated, which means that for each trip, a route is assigned from the origin to the destination;
5. The sensors are located on the map, and the trips that pass by the sensors are counted. Therefore, data from the synthetic sensors are extracted in this step for the set of random trips;
6. Synthetic sensor data from the previous step and the OD matrix of bus trips (output of the bus package) are given to the algorithm;
7. Finally, the algorithm outputs a set of trips, and the error can be calculated.

A set of 2827 random trips is generated in a sample validation run, and after going through the validation steps, the vehicle package outputs 2464 trips. In order to measure the precision of the method, the relative error is calculated:

(5) $$E=\frac{\left|2827-2464\right|}{2827}=12.8\%$$

**Figure 11:**
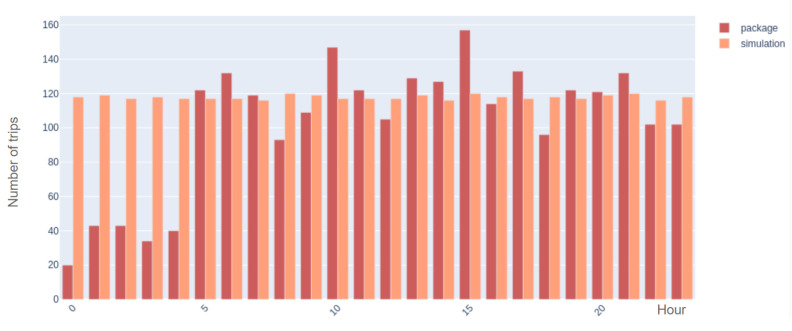
Comparison of hourly number of generated trips and the number of estimated trips by vehicle package.

Figure 11 presents the hourly distribution of the number of synthetic trips and the estimated number of trips by SUMO after calibration. It can be observed that the error is high from midnight until 5:00 a.m. The reason for this is the unavailability of bus data during these hours to initialize the algorithm.

However, if we only compare the output of the algorithm with the trip data after 5:00 a.m., starting from 2238 random trips, the algorithm estimates 2284 trips. The following calculation shows a considerable improvement in error.

(6) $$E=\frac{\left|2238-2284\right|}{2238}=2\%$$

### 11.3. Pedestrian Package

In this section, we explain the steps for validating the methodology used in the pedestrian package. It is worth mentioning that this package relies on a considerably few number of sensors, and the sensors only record the number of passersby but not their direction. Moreover, the presented solution is quite sensitive to the initial OD matrix distribution, which has to be given as external information.

**Figure 12:**
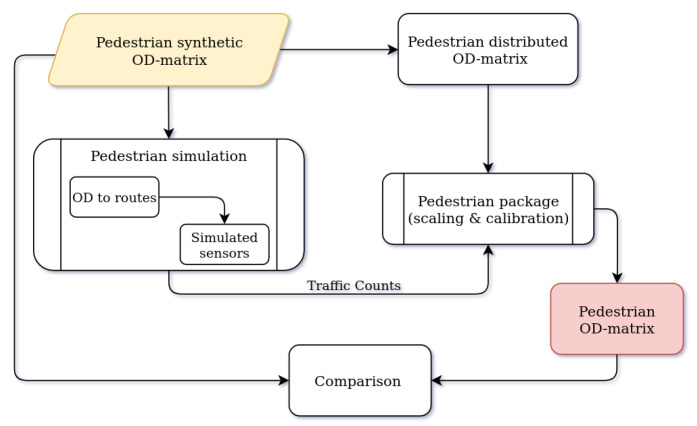
Validation process of the scaling and calibration model within the pedestrian package.

In order to validate our methodology in the lab environment, we generate synthetic input data for the package and analyze the output. The pedestrian package requires two sets of inputs: an initial distributed OD matrix and the sensor data for calibration. In the first step, a set of 100,000 random trips are generated, and the district-level OD matrix R of trips is extracted. Then, the routes are simulated using the SUMO simulator. In the next step, synthetic sensor data are extracted by considering the routes that pass the sensor locations. The initial distributed OD matrix D is also obtained by normalizing the OD matrix R. Finally, the package is run on synthetic input data, and the OD matrix X is generated. Figure 12 presents the validation process for the pedestrian package.

**Figure 13:**
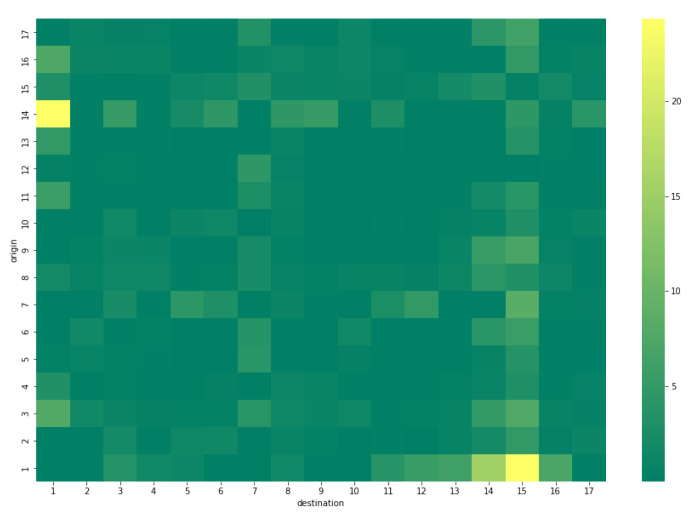
Relative error between the generated and estimated OD matrix of pedestrian trips.

For an evaluation of the package output, we computed the relative error between the number of generated and estimated trips for every pair of origin and destination. The result of this comparison is presented in Figure 13.

The total error of the pedestrian package with the synthetic data is computed in Equation (7).

(7) $$E=\frac{{∥R-X∥}_{2}}{{∥R∥}_{2}}\times 100=2.3\%.$$

The method implemented for estimating the pedestrian flow is highly sensitive to our understanding of high-level trip patterns and the likelihood of route choice in the network. With the presented validation, we showed that if the information is provided, we get a low error rate (Equation (7)). In another experiment, we generated a set of synthetic multi-modal bus and walking trips and followed the probability distribution of bus trips for the initial OD matrix, which yielded a relative error of 13.5% in the number of trips. High-quality input data can be obtained from any reliable source such as travel diaries or surveys. We can improve the accuracy of the probabilities by considering the population, places of interest, and network connectivity.

### 11.4. Field Test

**Figure 14:**
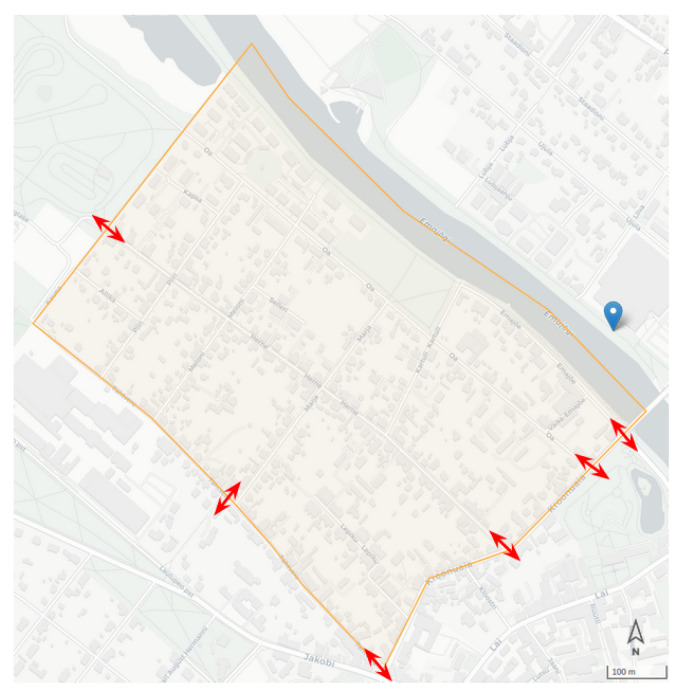
Testing area in the district of Supilinn and the locations of manual counts.

The field test was planned for 10 December 2021, from 08:00 to 10:00, in the district of Supilinn; it has an area of 0.48 km${}^{2}$ and a population of 2147. Figure 14 shows the district as well as the designated spots for collecting the manual counts. It is worth mentioning that there was no sensor inside the borders of the selected district, and the estimations relied on the measurements in the nearby districts.

In addition, on the selected date, the weather temperature dropped drastically to −19 ${}^{\circ }$C, which made our experiment an unplanned outlier and resulted in fewer walking trips as compared to the average. Moreover, due to the amount of snow, the number of cyclists dropped dramatically.

Excluding the public transport trips, in the same two-hour time interval, the system’s modal split estimation yields a relative error of 27% as compared to the modal split obtained by the actual counts. While our methodology is designed to capture district-level mobility, we do not expect very high accuracy in small spatial units. Table 2 shows the modal split obtained from the field experiment and the estimations from our system in the district of Supilinn.

## 12. Conclusions

In the era of digitalization and data sharing, there is great potential in enabling the transformation towards sustainable mobility. Data from different sources, especially from IoT devices within the cities, are central to the creation of smart cities and the future of urban mobility. Therefore, in this paper, we demonstrated the potential of real-time IoT data in providing information about urban mobility. Our platform is designed to ingest real-time data streams from IoT devices in order to produce an analysis of daily mobility shares in the city and provide the OD matrix distribution between the city’s districts.

The outcome and performance of the integrated algorithms in our platform showed encouraging results. The registered errors and the amount of data used by the sensors create a balance between quality and performance. The lab testing demonstrated errors varying between 2% and 13%, depending on the modules of Bike, Pedestrian, Vehicle, and Bus. Since the system depicts the mobility patterns in coarse granularity, to assess the system’s performance in the real environment, ground-truth counts had to take place in a large area of the city and for several hours. Nevertheless, with limited available resources, we conducted a field examination to test our system’s performance in a small district. No sensor exists in the selected district, and only a few sensors are available in the neighboring districts. The results showed satisfying outcomes, where the error varied between 0.1% and 15%, depending on the mode of transport.

However, there is still a window for refinement and increasing the system’s accuracy. The quality of input data can be improved by positioning more sensors in the city’s critical location, introducing more IoT devices capable of distinguishing between pedestrians and bicycles or able to provide a classification of vehicles. In addition, more sophisticated methods can be applied for estimations, such as through the introduction of an adaptive probabilities approach for optimizing the analysis process in simulation and calibration models. Alternatively, since information only exists for some specific road segments, exploring more advanced models such as the belief propagation [46] approach can be beneficial.

Finally, our motivation behind this created real-time system is to contribute to the foundations of smart cities, particularly from the mobility and transport aspects. Furthermore, it demonstrates the fruit of collaborating with our local city and the data’s potential in helping to understand and positively impact our citizens, environment, and local economy. Our vision is that the power of data and technology can be the key to innovation, opportunities, suitability, and a better future for humanity on our planet.

## Figures and Tables

**Table 1:** Data sources of the Modal Split pipeline.

Data Source	Packages	Type
Cumulocity ECO Sensor Measurements	Bike, Pedestrian	Dynamic
Cumulocity Thinnect Sensor Measurements	Pedestrian, Vehicle	Dynamic
Cumulocity AVC Sensor Events	Vehicle	Dynamic
Cumulocity Bus GTFS Events	Vehicle	Dynamic
Bewegen REST API	Bike	Dynamic
Ridango REST API	Bus	Dynamic
peatus.ee Bus Stop Information	Bus	Dynamic
Bicycle Station to District Map	Bike	Static
Tartu Districts Geo-data File	All packages	Static

**Table 2:** Modal split for the manual counts and the estimated counts for the district of Supilinn on 10 December 2021, from 8:00 to 10:00.

Mode of Transport	Modal Split Field Counts	Modal Split Estimation
Bike	1.2%	1.1%
Pedestrian	23.6%	38.9%
Vehicle	75.1%	59.9%

## Data Availability

Generated data in this study is available through a publicly accessible dashboard that does not issue DOIs. Publicly available datasets were generated in this study. This data can be found here: https://its.cs.ut.ee/modsplit/#odMatrix.
